# Primary hydatid cyst of the gallbladder: a case report

**DOI:** 10.1186/1752-1947-4-29

**Published:** 2010-01-29

**Authors:** Avdyl Krasniqi, Dalip Limani, Lumturije Gashi-Luci, Gazmend Spahija, Ismail A Dreshaj

**Affiliations:** 1 University Clinical Center of Kosovo, Division of Abdominal Surgery, Medical School University of Prishtina, Prishtina, Republic of Kosovo; 2 University Clinical Center of Kosovo, Institute of Pathology, Medical School University of Prishtina, Prishtina, Republic of Kosovo; 3 University Clinical Center of Kosovo, Department of Anesthesiology and Reanimation, Prishtina, Republic of Kosovo; 4 University Hospitals & Case Medical Center, Cleveland, Ohio, USA

## Abstract

**Introduction:**

Echinococcosis, or hydatid disease, is endemic in some regions of the world, and has been a common pathology of surgical wards in Kosovo. Primary hydatid cyst of the gallbladder is an unusual and very rare localization of hydatid disease. So far, only five cases that fulfill the criteria of primary gallbladder hydatidosis have been published in the English medical literature.

**Case presentation:**

We report a case of a 39-year-old Kosovan Albanian woman referred to the Abdominal Surgery Division of the University Clinical Center of Kosovo for "a calcified hydatid cyst of the liver with gallbladder involvement". Her history was significant for chronic right upper quadrant pain, characterized as intermittently colicky pain, accompanied by nausea. The patient underwent right subcostal laparotomy. Intra-operatively, a calcified primary hydatid cyst of the gallbladder was found. Its pericyst was tightly attached to the liver. Complete pericystectomy with cholecystectomy followed. The histopathology confirmed the presence of calcified hydatid cyst of the gallbladder, and that the cyst had developed entirely extra-mucosally. Five year follow-up showed no recurrence of disease.

**Conclusion:**

Primary hydatid cyst of the gallbladder is a very rare clinical entity. Accurate preoperative diagnostic localization is not always easy, particularly in centers with limited diagnostic tools.

## Introduction

Hydatid disease is a zoonotic infection caused by larval stages of dog tapeworms belonging to the genus Echinococcus (family taeniidae) and is also referred to as echinococcosis [1]. Three broad morphological forms of echinococcosis are recognized clinically. Human cystic echinococcosis caused by *E granulosus *is the most common presentation and probably accounts for more than 95% of the estimated 2-3 million annual worldwide cases [2]. This disease continues to be a substantial cause of morbidity and mortality in many parts of the world [1]. Hydatidosis is endemic in Mediterranean countries and other sheep and cattle-raising regions [3]. In Kosovo, although there is no exact data about the incidence of human cystic echinococcosis, liver and lung hydatid cysts continue to be a very common pathology of surgical wards [4]. The liver (70-80%) and lungs (15-25%) are the most frequent locations for echinococcal cysts while occurrence in other sites is very rare [3-6] and the real incidence of extra hepatic cysts is not known [5].

Primary hydatid cyst of the gallbladder is an extremely rare entity [6-8]. There are reports of the gallbladder daughter cysts secondary to liver cysts [9]. Patients with primary hydatid cyst of the gallbladder are those with no previous history of hydatid disease and with no other cysts found at the time of surgery [6]. In a recent review of the literature through the Medline database, we found that in the English language only five cases have been reported. by Safioleas*et al*. (2004), Rigas*et al*. (1979); Wani*et al*. (2005), and Raza*et al*. (2003) [5-8]. Two more cases were reported in Slavic languages [10,11]. The aim of this case report is to highlight the diagnostic features, routes of dissemination and treatment options of this rare clinical entity.

## Case presentation

**Figure 1:**
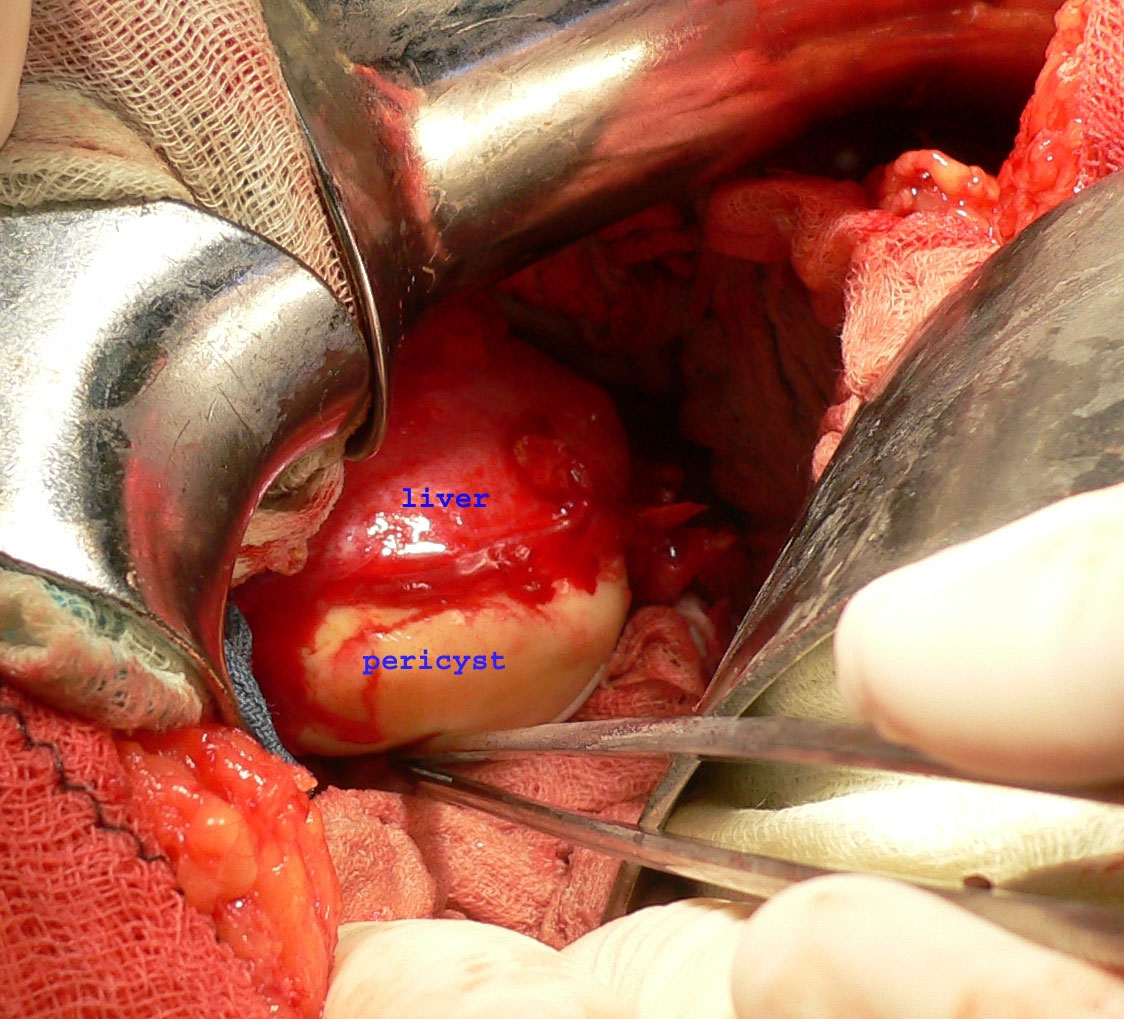
**Intraoperative view: Outer side of calcified hydatid cyst tightly attached to the liver**.

**Figure 2:**
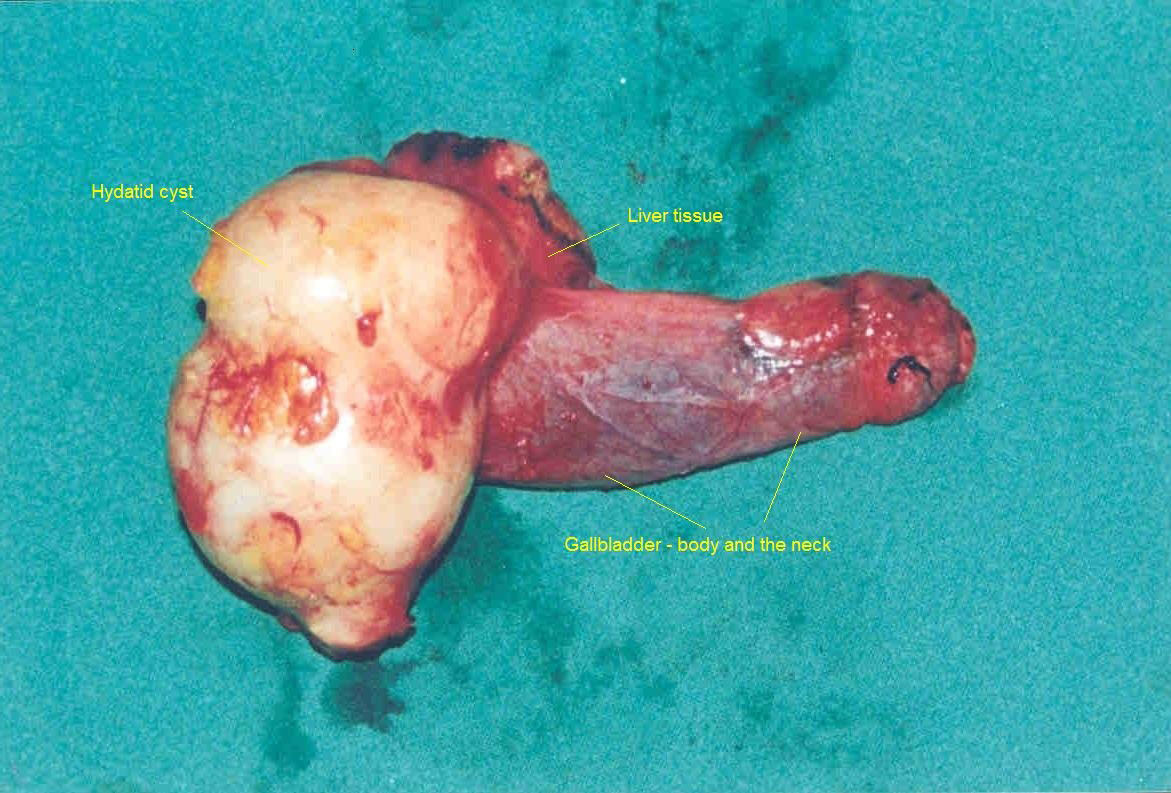
**The outer side of the removed cyst together with the gallbladder and a small part of the liver**.

**Figure 3:**
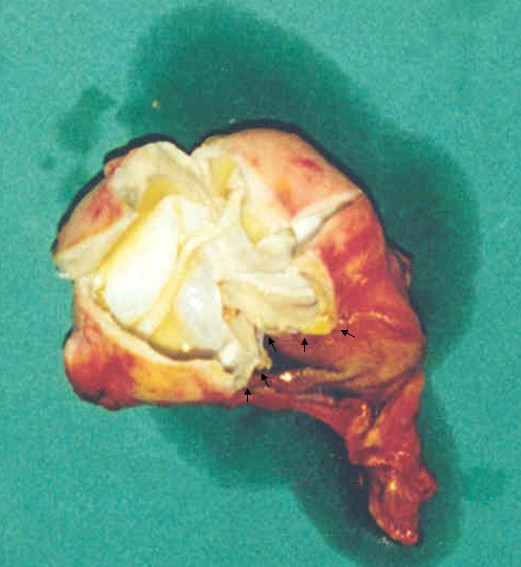
**The inner side of the cyst developed entirely extramucosally**.

**Figure 4:**
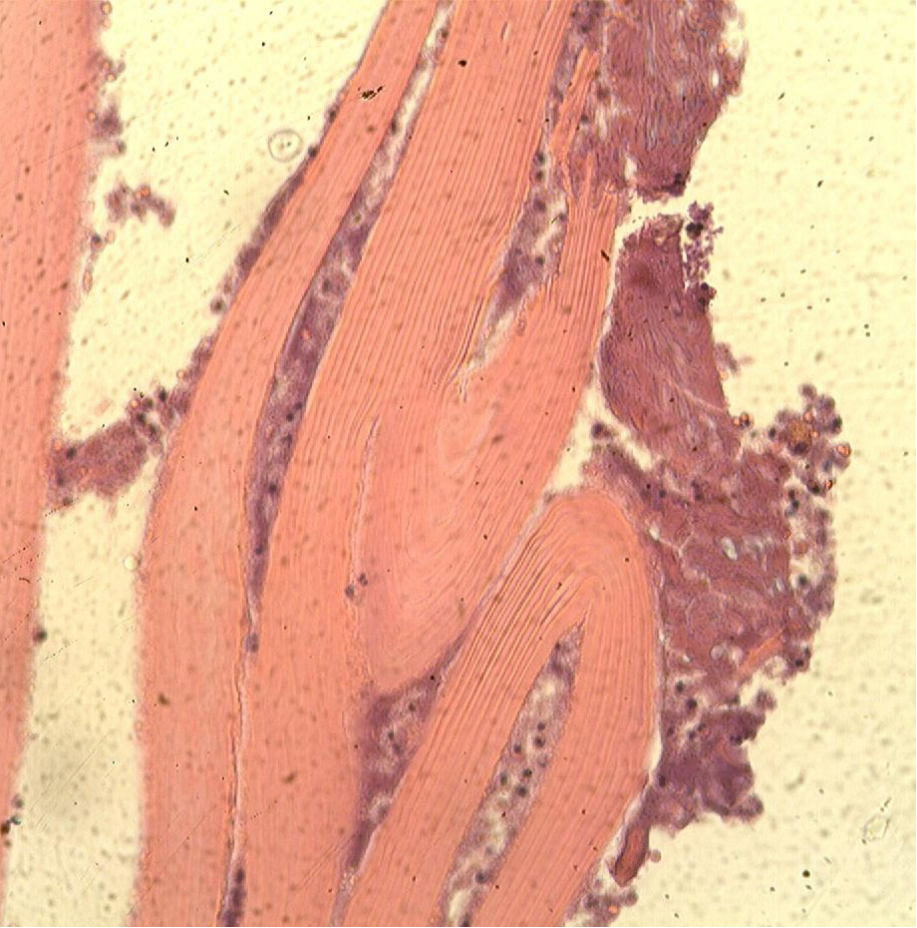
**Histopathologic specimen**.

A 39 year old Kosovan Albanian woman was referred to the Division of Abdominal Surgery at the University Clinical Center of Kosovo for an 18-month history of chronic (intermittently colicky) pain in the right upper quadrant, often accompanied by nausea. There was no history of jaundice. She had been treated by her primary care physician with antispasmodics, H2 receptor blockers and antibiotics, without resolution. On admission, physical examination showed no abnormal abdominal findings except mild tenderness in the right upper quadrant. Routine blood tests such as CBC, renal and liver panel proved unremarkable. Chest x-ray showed no signs of cardio-respiratory disease. Plain radiograph of the abdomen showed a calcified opacity at the level L2-L3 vertebrae on the right. The diagnosis, supported by ultrasound and computed tomography, was a calcified hydatid cyst of the liver with involvement and deformation of the gallbladder; the architecture and the size of biliary ducts were normal. The patient underwent right subcostal laparotomy. Intra-operatively, a calcified primary hydatid cyst of the fundus and body of the gallbladder was found with its pericyst attached to the liver (Figure 1). The inflammatory response of the liver tissue against the cyst was extensive and formed the structural part of the posterior wall of the pericyst. Complete pericystectomy along with cholecystectomy was performed. No other cysts were found during careful exploration of the peritoneal cavity. On opening the gallbladder, a calcified hydatid cyst (dimensions 7 cm × 5 cm) was found, located in the body and fundus of the gallbladder (Figure 2). Macroscopically, the hydatid cyst was part of the gallbladder. The cyst had reduced severely the lumen of the gallbladder and had grown entirely submucosally (Figure 3). The histopathology confirmed the presence of calcified hydatid cyst of the gallbladder (Figure 4). The patient's postoperative course was uneventful and she was discharged in good condition on the seventh postoperative day. She received two 21-day courses of oral Albendazol 400 mg/day with 14 days pause in between. At five-year follow up, she has had no recurrence of hydatid disease.

## Discussion

Hydatid disease is a common clinical pathology in many parts of the world. There are two clinical forms of this disease: cystic hydatidosis caused by *Echinococcus granulosus *and alveolar hydatidosis caused by *Echinococcus multilocularis*. The main species pathogenic for humans in Mediterranean and Southern European countries is Echinococcus granulosus [1]. There has been no reported case of alveolar hydatidosis in Kosovo [4]. Infection begins with the ingestion of tapeworm eggs, which in the human intestine hatch into embryos that penetrate the small bowel mucosa, enter venules and travel via portal circulation to the liver. Hydatid cysts most often develop in the liver. However when embryos pass through this first filter, the second most frequent location is the lung. Hydatid cysts can occur anywhere in the body [3-7,12].

Unusual locations of hydatid cysts support the hypothesis that beside portal circulation, the echinococcus embryos can spread via other routes, such as the lymphatic system [6,8,12], the biliary tract [7,13] and/or by dissemination of daughter cysts into peritoneal or other cavities with the rupture of the primary cyst [3,13]. Because of the small number of cases reported, opinions about the pathogenesis of the primary gallbladder hydatid cysts are divided depending on the location of the cyst: in the lumen of the gallbladder or on the external surface [6-8,13]. In two case reports, Cangiotti *et al*. (1994) [13] and Raza *et al*. (2003) [7] found cysts inside the gallbladder and described them as a result of brood capsules dissemination through the biliary tract (i.e. through the cystic duct). Safioleas et al. (2004) [6] reported findings in three cases and remphasizes the idea of Rigas et al. (1979) [8] of lymphatic rather than biliary spread in primary gallbladder hydatid disease. In our case, the hydatid cyst was larger than it was described in previous reports [6-8,13]. Macroscopic as well as microscopic examination of the removed formation after surgery, showed very clearly that the hydatid cyst narrowed severely the lumen of the gallbladder, but that it had grown entirely extra-mucosally (Figure 3). In this case, therefore, transport of oncospheres from the intestine to the gallbladder is more likely to have occurred by lymphatic circulation. However, that should be confirmed in a larger number of patients.

Pain, midabdominal discomfort and dyspepsia were the main symptoms in all reported cases of primary hydatid cyst of the gallbladder. Neither jaundice nor anaphylactic reaction were noted in any cases [5-7]. Chronic upper abdominal low intensity pain, nausea and intermittent biliary colic were the main complaints of our patient. Imaging diagnostic tools such as ultrasound and computed tomography are very helpful in the diagnosis of hydatid cysts [3,4,6,9,14]. Due to the anatomic proximity of the gallbladder to the liver, exact localization of primary gallbladder hydatid cysts is not always easy preoperatively, particularly in centers with limited resources. The exact site of extra hepatic abdominal hydatidosis in some cases is confirmed only intra-operatively [4-6], as in this case. The initial ultrasound and computed tomography scans described the cystic lesion as a hydatid cyst of the liver with involvement of the gallbladder, only to have the diagnosis corrected during surgery. This is the first case of a primary gallbladder hydatid cyst in our experience of 241 patients with liver cystic hydatidosis [15]. Gallbladder hydatid cysts should be differentiated from liver hydatid cysts and other extra hepatic cystic lesions [4-6]. Liver hydatidosis has a long asymptomatic period of cystic growth [1], whereas in the primary gallbladder hydatid cyst, biliary symptoms begin earlier, and diagnostic imaging indicates smaller cysts with deformation of the gallbladder [6].

Surgery is the preferred treatment for hydatid disease. The goal is the eradication of the parasite without spillage of the cyst content. In liver hydatididosis, complete pericystectomy is not always possible, and therefore partial perycystectomy is the most frequent surgical approach [3,4]. Successful total pericystectomy with cholecystectomy was performed in all reported cases [6-8,10]. We too performed perycystectomy with cholecystectomy. Postoperative recovery was uneventful. We found, as did other authors [5-7], that in primary hydatid cyst of the gallbladder, radical excision is easier than it is in liver hydatidosis [3,4]. In case of exuberant immune reaction of liver tissues against the calcified pericyst of gallbladder, dissection should be done very carefully to avoid injuring biliary ducts that are in the proximity to the gallbladder bed.

## Conclusions

Primary hydatid cyst of the gallbladder is a very rare clinical entity. Accurate site diagnosis was not made pre but intra-operatively. In our experience of 241 patients treated surgically for liver hydatididosis, only one patient (0.4%) was found to have primary gallbladder hydatid cyst. Careful pericystectomy with cholecystectomy is the procedure of choice for radical excision of primary hydatid cysts. Compare to liver hydatidosis, the gallbladder primary hydatid cyst has different spread routs of parasite embryos and a better prognosis due to earlier manifestation of symptoms leading to earlier treatment.

## List of abbreviations

CBC: Complete blood count; US: Ultrasound; CT: Computerized tomography.

## Consent

Written informed consent was obtained from the patient for publication of this case report and accompanying images. A copy of the written consent is available for review by the Editor-in-Chief of this journal.

## Competing interests

The authors declare that they have no competing interests.

## Authors' contributions

AK and DL performed the surgery and paper writing. LGL performed the histological examination of the specimen. GS conducted anesthesia and searched the literature. ID helped with conservative therapy and paper writing.

All authors have read and approved the final manuscript.
